# Contribution of individual and cumulative frailty-related health deficits on cardiac rehabilitation completion

**DOI:** 10.1186/s12877-022-03624-0

**Published:** 2023-01-20

**Authors:** Troy Hillier, Evan MacEachern, Dustin S. Kehler, Nicholas Giacomantonio

**Affiliations:** 1 Faculty of Medicine, Dalhousie University, Halifax, NS Canada; 2 School of Physiotherapy, Faculty of Health, Dalhousie University, Halifax, NS Canada; 3 Division of Geriatric Medicine, Department of Medicine, Dalhousie University, Halifax, NS Canada; 4 Division of Cardiology, Department of Medicine, Dalhousie University, Halifax, NS Canada

**Keywords:** Cardiac rehabilitation, Frailty, Non-traditional, Cross-sectional

## Abstract

**Background:**

Despite the high burden of frailty among cardiac rehabilitation (CR) participants, it is unclear which frailty-related deficits are related to program completion.

**Methods:**

Data from a single-centre exercise- and education-based CR program were included. A frailty index (FI) based on 25 health deficits was constructed. Logistic regression was used to estimate the odds of CR completion based on the presence of individual FI items. The odds of completion for cumulative deficits related to biomarkers, body composition, quality of life, as well as a composite of traditional and non-traditional cardiovascular risk factor domains were examined.

**Results:**

A total of 3,756 individuals were included in analyses. Eight of 25 FI variables were positively associated with program completion while 8 others were negatively associated with completion. The variable with the strongest positive association was the food frequency questionnaire score (OR 1.27 (95% CI 1.14, 1.41), whereas the deficit with strongest negative association was a decline in health over the last year (OR 0.74 (95% CI 0.58, 0.93). An increased number of cardiovascular deficits were associated with an increased odds of CR completion (OR per 1 deficit increase 1.16 (95% CI 1.11, 1.22)). A higher number of traditional CR deficits were predictive of CR completion (OR 1.22 (95% CI 1.16, 1.29)), but non-traditional measures predicted non-completion (OR 0.95 (95% CI 0.92, 0.97)).

**Conclusion:**

A greater number of non-traditional cardiovascular deficits was associated with non-completion. These data should be used to implement intervention to patients who are most vulnerable to drop out to maximize retention.

**Supplementary Information:**

The online version contains supplementary material available at 10.1186/s12877-022-03624-0.

## Introduction

The efficacy of exercise-based cardiac rehabilitation (CR) for reducing complications related to cardiovascular disease has been consistently demonstrated [1–4]. A recent Cochrane review observed a significant decrease in the risk of cardiovascular mortality, and overall hospitalization rates amongst individuals who completed center-based CR compared to those who did not [1]. A recent meta-analysis reported that CR has a positive impact on both physical and emotional health-related quality of life, demonstrating that health factors beyond those related to cardiovascular health alone can be improved with CR participation [2]. Despite the demonstrated benefits of CR, attendance rates are variable (estimated at approximately 34% in North America [5]), and improvement of attendance is a priority [4, 6, 7]. Completion of CR is important in health improvement – and is a cost-effective intervention [8] – for individuals with cardiovascular disease [3, 4, 9–14], as previous research has demonstrated little to no positive impact for participants who do not complete the majority of their prescribed program [15, 16].

Patients entering CR are now older and more likely to be burdened with other health problems not necessarily related to their referring diagnosis [17]. Frailty can conceptualize the variability in the accumulation of health problems as people age. That is, characterizing frailty allows for the comparison of vulnerability to health complications between individuals of similar ages [18]. In the context of cardiovascular disease, frailty is important as a marker of overall health status [19–21], which makes it relevant to patients who are referred to CR [22–24]. Higher frailty levels at CR admission have recently been demonstrated to be significantly associated with non-completion of CR, which will affect patient prognosis [25]. Additionally, research has shown that patients who were the most frail at the onset of CR also gained the most from completion of the program [10, 12, 14, 25]. A recent study reported associations between smoking status, employment, and marital status with CR noncompletion when controlling for frailty [25]. However, no existing study has investigated the relationship between individual health deficits and program completion when controlling for levels of frailty. If individual health deficits that contribute to frailty are related to CR completion, patients with increased deficits on these parameters could be targeted with additional intervention to promote completing CR. The objective of this study was to determine which patient characteristics and age-related health deficits are important in patients completing CR. We wish to elicit further insight into the relationship between frailty and CR completion, therefore identifying patients who may benefit most from targeted interventions to increase program retention.

## Methods

### Data source and variables

Patients were eligible for CR if they experienced an acute cardiovascular event, including coronary artery disease, myocardial infarction, percutaneous coronary intervention, coronary artery bypass graft or valve surgery, heart failure or a combination of other diagnoses with few referrals (e.g., arrhythmia, heart transplant). Patients were excluded if they had a critical illness (e.g., for renal failure, cancer) requiring other treatments or another condition that would preclude them from participating in the program, such as severe stroke. Patients were also excluded if a frailty index (FI) could not be calculated. A total of 4,004 patients enrolled in a 12-week exercise and education-based CR program between 2005–2015 were eligible for the current study [25].

Routinely collected CR data from the nurse practitioner, dietician, and physiotherapist staff were used in this study. Clinical data were subsequently entered into a research database by a research assistant that were used for the present study. Participant characteristics included admission demographics consisting of age in years, sex (% female), smoking status (non-smoker, ex-smoker, current smoker), marital status (Divorced/separated, widowed, single, married/living with partner), education (< grade 12, grade 12/GED, community college, bachelor’s degree, post secondary education), employment (disability, unemployed, part-time, full-time, retired, other), beta-blocker and lipid lowering medication use, and referring diagnosis (see above list for eligibility into CR).

Frailty was measured with a 25-item FI at admission using a guidelines-based approach and previously used in the CR cohort [25, 26]. The following items were included in the FI: 1) cardiovascular risk factors including triglycerides, total cholesterol, low density lipoprotein, and high-density lipoprotein, fasting blood glucose, systolic and diastolic blood pressure, resting pulse rate, pulse pressure, and mean arterial pressure; 2) cardiovascular symptoms with the New York Heart Association functional class system; 3) cardiovascular fitness from an exercise stress test; 4) quality of life with the SF-36 questionnaire to capture physical, mental, social and general health domains; 5) body composition with body mass index, waist circumference, and bioelectrical impedance (to measure percent fat and fat free mass); and 6) diet quality with the Food Frequency Questionnaire. The FI is a ratio of the total accumulated health deficits across multiple physiologic systems (scores range from 0 to 1; a higher score indicates a higher frailty level). A valid FI was determined if participants had less than 30% missing data for the FI constituents [27]. An in depth description of the FI and its constituents was previously published [25]. FI distribution by program completion can be found in Table 1. The primary outcome measure for the current study was CR program completion; defined as attendance of at least 65% of sessions, coupled with the completion of a discharge assessment [25].

**Table 1:** Distribution of demographic variables for participants in CR stratified by completion

	Completed CR		
	No (*n* = 1,335)	Yes (*n* = 2,669)	*p*
Age	60.6 (11.2)	62.4 (10.7)	< 0.001
Sex			0.03
Female	369 (28)	654 (25)	
Frailty			< 0.001
< 0.2	116 (10)	448 (17)	
0.2–0.3	238 (21)	715 (27)	
0.3–0.4	290 (26)	791 (30)	
0.4–0.5	237 (21)	462 (17)	
> 0.5	232 (21)	227 (9)	
Missing	222	26	
Smoker			< 0.001
Non-Smoker	356 (27)	865 (32)	
Ex-Smoker	761 (57)	1585 (59)	
Current Smoker	218 (16)	219 (8)	
Marital Status			< 0.001
Divorced/separated	158 (12)	212 (8)	
Widowed	105(8)	170 (6)	
Single	129 (10)	179 (7)	
Married/living with partner	943 (71)	2108 (79)	
Beta blocker			0.004
Yes	1016 (76)	2136 (80)	
Lipid lowering medication			< 0.001
No	341 (26)	472 (18)	
Yes	994 (74)	2197 (82)	
Education			< 0.001
< Grade 12	343 (26)	505 (19)	
Grade 12/ GED	245 (18)	482 (18)	
Community College	385 (29)	839 (31)	
Bachelor's Degree	241 (18)	542 (20)	
Post-Secondary	121 (9)	301 (11)	
Employment			< 0.001
Disability	141 (11)	140 (5)	
Unemployed	63 (5)	48 (2)	
Part time	76 (6)	150 (6)	
Full time	297 (22)	539 (20)	
Retired	526 (39)	1297 (49)	
Other	232 (17)	495 (19)	
Diagnosis			0.002
Coronary artery disease	354 (27)	731 (27)	
Percutaneous coronary intervention	204 (15)	392 (15)	
Cardiac Surgery	212 (16)	521 (20)	
Heart Failure	118 (9)	184 (7)	
Myocardial infarction	367 (27)	736 (28)	
Other	80 (6)	105 (4)	

Age is represented as mean (SD), all other variables are categorical and are represented as frequency (%). *P*-values presented represent t-tests for continuous variables (age), and chi-squared tests for categorical variables

### Cardiac rehabilitation program

The CR program was a phase two traditional center- and group-based 12-week behaviour change intervention, with sessions focused on education (1 session/week) and exercise (up to 2 sessions/week), each lasting approximately 60 min. Exercise sessions were structured as follows: a 10-min warm-up, 40 min of aerobic training, and 10 min of resistance training. The goals of the program were to reduce CVD risk factors by incorporating exercise, improving diet, and optimizing medical management. Patients were given an exercise prescription for both group-based sessions and for their home-based program. These prescriptions were individually tailored to the patient [25].

The 12-week CR program provided patients with a multidisciplinary healthcare team, consisting of a CR program lead, nurse practitioner, nurses, physiotherapist, and dietician, guided by the cardiac medical director [25]. Upon admission to CR, personal CR goals were established using the SMART-framework [28] and were facilitated with assistance from CR staff [25]. A graded exercise test using a cycle ergometer or treadmill was used to determine a patients’ maximum metabolic equivalent (MET) level achieved to aid in exercise prescription. Examples of patient goals in CR include weight loss (i.e. lowering BMI, reducing waist circumference), improving physical fitness (i.e. returning to previous sport/hobbies, improving physical strength), and improving cardiovascular profile (i.e. lowering cholesterol, control/manage diabetes, smoking cessation, and hypertension management) [25].

### Analysis

Demographic variables are presented as frequency (percent) or mean (standard deviation) for categorical and continuous variables, respectively. Demographics, and program completion across levels of frailty were compared using logistic regression. Individual variables contributing to the FI were entered into multivariable models controlling for age, sex, education, marital status, employment, smoking status, and referring diagnosis. Based on individual deficits, odds ratios for program completion and 95% confidence intervals were estimated. All variables were standardized such that the odds ratios presented represent a 10% change in each of the individual FI deficits. The cumulative effect of deficits in domains related to cardiovascular health, body composition, and quality of life categories were also investigated for eligible participants (Supplemental Table S1). The categorizations were as follows, cardiovascular (systolic blood pressure, diastolic blood pressure, mean arterial pressure, pulse pressure, pulse rate, cholesterol level, triglyceride level, low-density lipoproteins, high-density lipoproteins, blood glucose, and NYHA functional category); body composition (BMI (body mass index), waist circumference, fat-free mass, food frequency questionnaire); quality of life (METs, SF-36 physical function, SF-36 mental health, SF-36 role physical, SF-36 general health, SF-36 energy, SF-36 role physical, SF-36 change in health past year, SF-36 bodily pain). The cut-offs indicating a deficit for this analysis were published previously [25] (Supplemental Table S2); however, for non-binary categorizations used in the previous paper, categories were appropriately combined for analysis. That is, if a variable consisted of 4 categories, the lower two were combined to signify the presence of a deficit and the higher two combined to signify the absence of a deficit. For example, NYHA categories three and four were combined to represent the presence of a deficit for that variable. Where necessary, categories were collapsed such that no individual category contained less than 5% of the overall sample. Additionally, cumulative traditional cardiovascular (cardiovascular plus METs) and non-traditional cardiovascular (SF-36 variables and body composition variables) deficits were investigated. In these analyses, cumulative deficits was defined as the sum of the number of deficits an individual had in each previously described category. Covariate selection for final models was performed using backward stepwise selection, with a liberal *p*-value (0.15) [29]. Effect modification of individual frailty deficits with program non-completion was investigated by age, sex, referring diagnosis, and baseline frailty. All analyses were completed using SAS 9.4 (SAS Institute, Cary, NC, USA).

### Ethical considerations

This study was an analysis of data collected as part of a CR program. Nova Scotia Health Authority privacy policies were followed during the conduction of the study. Thus, the risk for harming individual patients was minimal. No known privacy breaches occurred during this study.

## Results

A total of 4,004 individuals were eligible, and 3,756 had sufficient information to allow for the calculation of an FI at baseline. The distribution of demographic variables and baseline frailty levels stratified by CR completion are displayed in Table 1. Non-completers of CR were slightly younger, more frail, less likely to be married, less educated, and more likely to be working than those who completed CR.

**Fig. 1:**
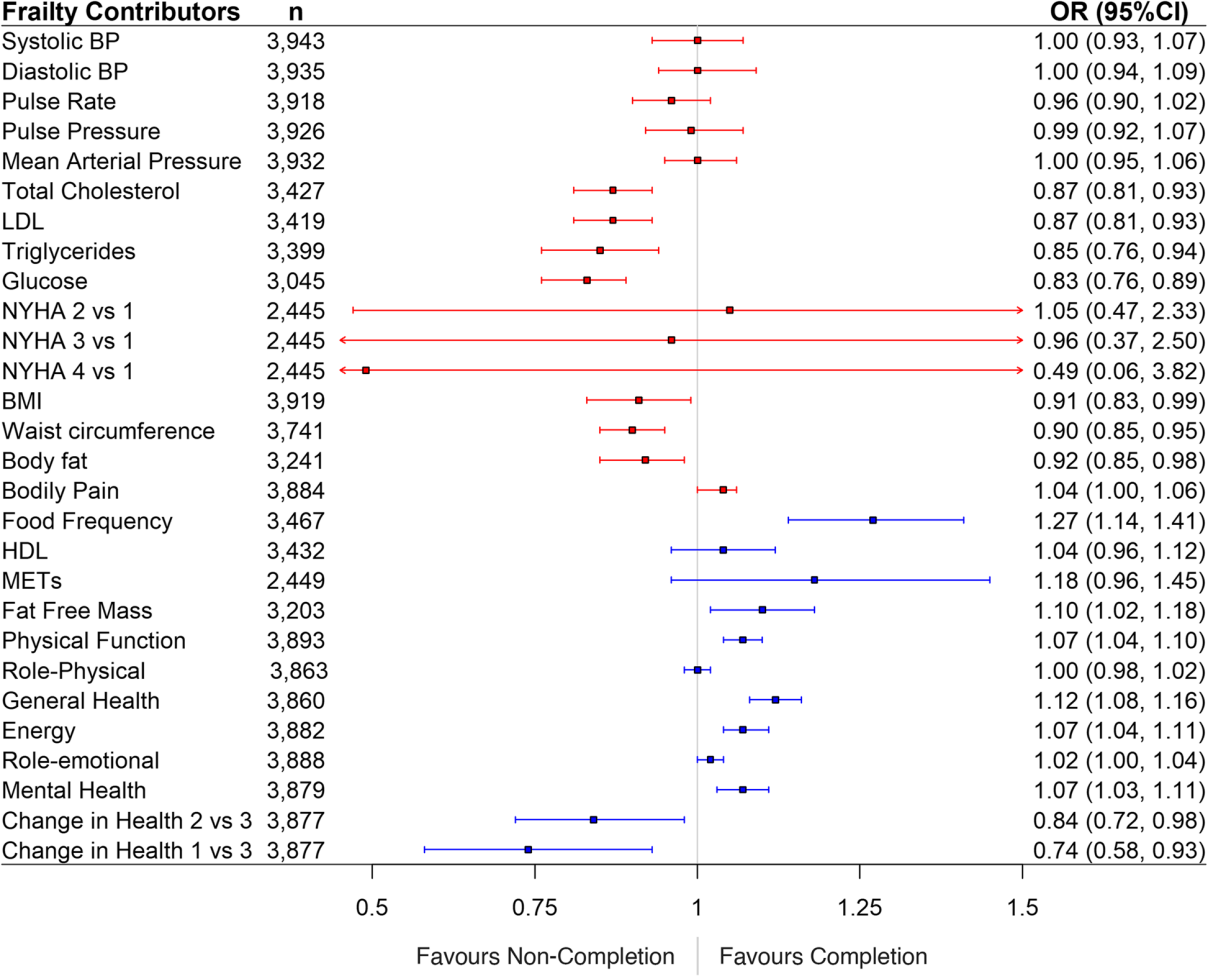
Forest plots of ORs (95% CI) for individual variables; odds ratios displayed represent a 10% change in the variable for all but NYHA scores and change in past year health. Odds ratios less than one indicate decreased likelihood of program completion, whereas those above one are indicative of increased completion. Confidence intervals displayed in blue signify variables where an increased value is favourable, whereas increases in those in red are unfavorable

The ORs for completion of CR for each of the variables contributing to the FI are displayed in Fig. 1. Of the twenty-five variables, sixteen deficits were independently associated with completion of the CR program. Ten percent increases in total cholesterol, low density lipoprotein, triglycerides, glucose, BMI, waist circumference, body fat, and past year changes in health were associated with a decrease in the odds of program completion (Fig. 1). Additionally, ten percent increases in physical function, bodily pain, general health, role emotional, and mental health domains from the SF-36, along with food frequency questionnaire responses, and fat free mass were associated with an increased likelihood of CR completion.

**Fig. 2:**
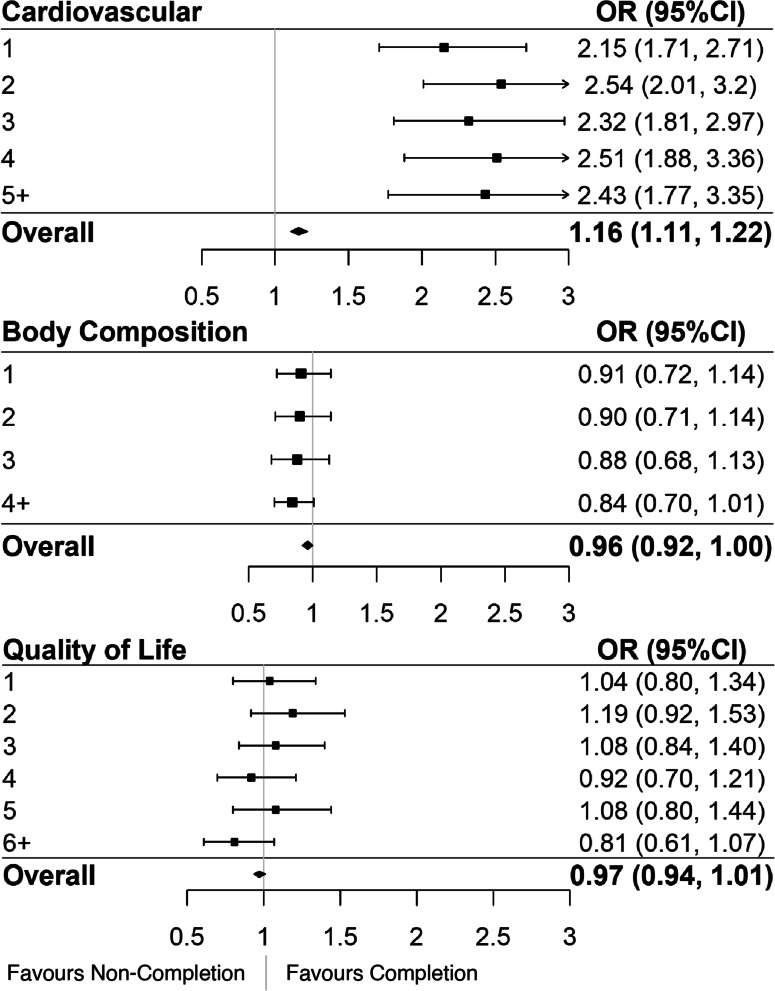
Accumulated deficits for each category ((**A**) cardiovascular health, (**B**) body composition, (**C**) quality of life) of variables contributing to frailty. Odds ratios less than one indicate increased likelihood of program non-completion, whereas those above one are indicative of increased completion

Further analyses aimed to identify whether a greater number of cumulative health deficits from the cardiovascular, body composition, and quality of life domains were associated with CR non-completion (Fig. 2). An increased number of deficits in the cardiovascular domain at CR admission were associated with a higher odds of completion, with a significant trend existing for each additional deficit resulting in an odds ratio for non-completion of 1.16 (95% CI 1.11, 1.22). Only a small difference in odds ratios for program non-completion existed for each individual number of deficits in this domain with point estimates ranging from 2.15–2.54 (Fig. 2). In the body composition domain, the odds of completion decreased for each subsequent deficit (OR = 0.96 (95% CI 0.92, 1.00)). That is, an individual categorized as having 3 of the possible 5 deficits in this domain has a significantly lower odds of completing to program as someone with only 2 deficits. However, no individual body composition deficits were associated with CR completion (Fig. 2). There were no significant associations between quality-of-life deficits and CR completion (Fig. 2).

**Fig. 3:**
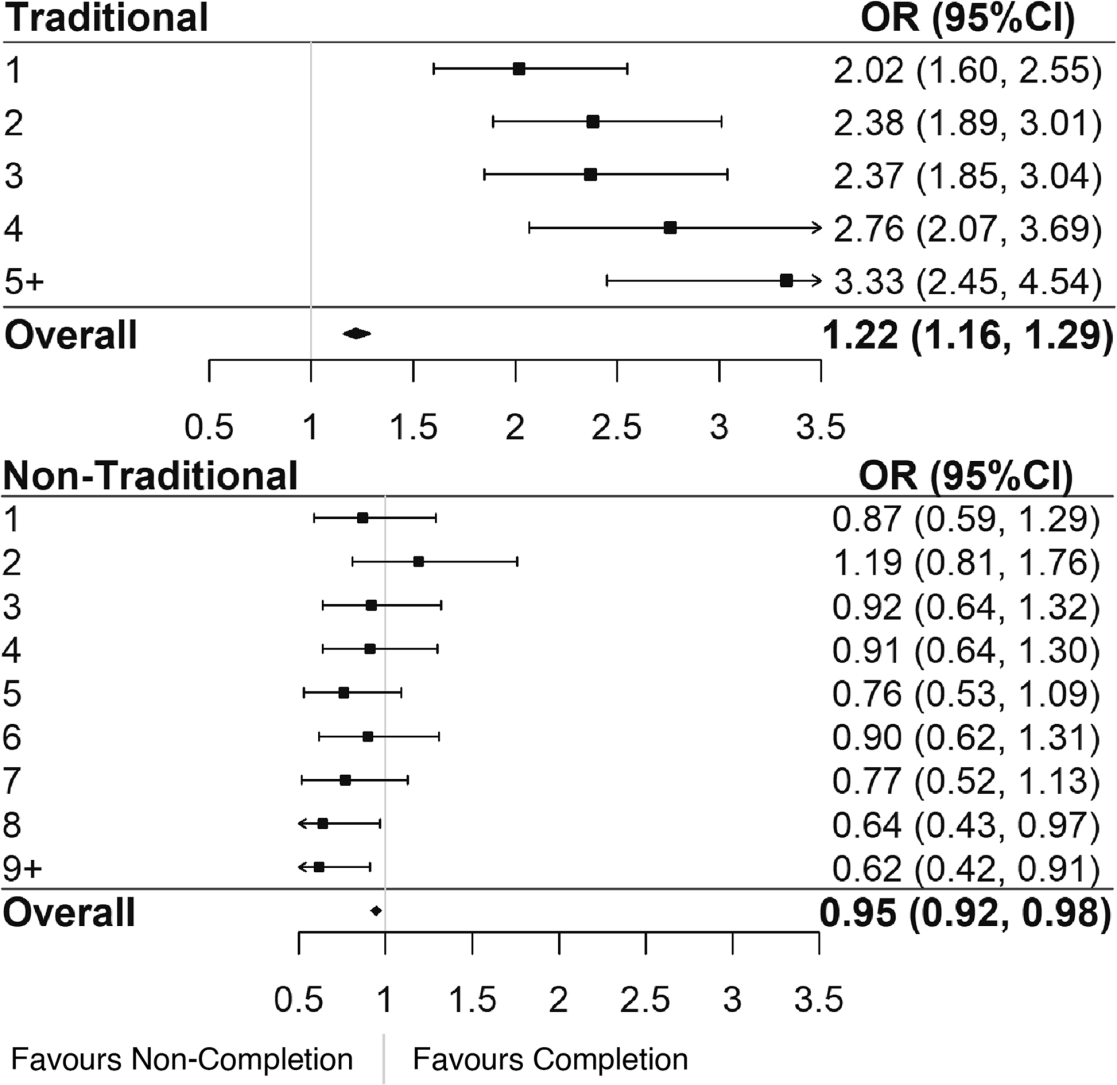
Accumulated deficits for (**A**) traditional cardiovascular variables, (**B**) non-traditional cardiovascular variables. Odds ratios less than one indicate increased likelihood of program non-completion, whereas those above one are indicative of increased completion

FI contributors were further categorized into traditional and non-traditional cardiovascular health measures (Fig. 3). Variables included in traditional and non-traditional CV deficit categories were previously discussed, and can be found in Supplemental Table S1. As participants accumulated more traditionally measured cardiovascular deficits, their likelihood of program completion increased (OR per deficit: 1.22 (95% CI 1.16, 1.29)) (Fig. 3). In the traditional cardiovascular health outcome domain, the odds ratios for program non-completion were significantly above 1, which indicates a higher likelihood of completion. Further, these odds ratios trended in the direction of individuals with the largest number of deficits (5 +) in this domain having the highest odds of completion (*p* < 0.001). In the non-traditional cardiovascular health domain, individuals with 8 and 9 + health deficits had significantly decreased odds of completion compared to those with no deficits (OR 0.64 (95% CI 0.43, 0.97) and 0.62 (95% CI 0.42, 0.91) respectively) (Fig. 3). Additionally, as non-traditional deficits were accumulated, the likelihood of program completion decreased significantly (OR per deficit 0.95 95% CI 0.92, 0.97). That is, individuals with a greater number of deficits in the non-traditional domain, especially those with 8 + deficits, are significantly less likely to complete CR.

Sensitivity analyses were conducted to discern whether any effect modification of the previously described relationships between individual FI deficits and CR completion existed by age, sex, baseline frailty, and referring diagnosis (Supplemental Table S3). The only significant interactions identified were between SF-36 role emotional with baseline frailty and referring diagnosis, as well as pulse pressure and baseline frailty (Supplemental Table S3).

## Discussion

We aimed to identify individual health deficits contributing to frailty that are associated with CR completion. Of the 25 deficits examined, 16 were independently associated with CR completion. A 10% increase in individual deficits associated with lower rates of completion included biomarkers (serum LDL, triglycerides, glucose, and total cholesterol), and body composition variables (BMI, body fat percentage, and waist circumference). Further, patients who identified a negative change in their health (according to the SF-36) in the last year were less likely to complete CR. Higher scores on quality of life measured by the SF-36 (body pain, physical function, general health, energy, role emotional, and mental health), as well as fat free mass, and the food frequency questionnaire were associated with an increased odds of CR completion. A 10% increase in the food frequency questionnaire score was associated with the greatest odds of CR completion. One potential reason for this could be that those individuals who were more concerned eating a healthy diet were also most concerned with their physical health, therefore resulting in a higher odds of CR completion. These findings demonstrate that while traditionally measured factors such as biomarkers and body composition have an impact on the likelihood of completing an exercise-based CR program, so do non-traditional measures which estimate quality of life. A total of 7 unique quality of life variables were associated with CR completion. That is, a patient’s self-reported quality of life is an important aspect to focus on when identifying individuals who are at risk of drop out from CR. Quality of life-related deficits could be addressed by the addition of mental health interventions for these patients with the aim of having positive impacts on self-image, as well as physical fitness. Overall, the current investigation identifies health deficits that should be focused on by program administrators to ensure maximum retention in CR programs. In the future, patients with deficits identified by this research should be focused upon for maximal program retention.

Even though we have demonstrated that older age was associated with greater CR completion rates, frailty is an independent risk factor for failure to finish the program [9]. Here, we expand on our analysis of the association of frailty with CR program completion. We investigated whether participants who have accumulated more deficits in specific frailty domains (cardiovascular, body composition, quality of life, and traditional vs. non-traditional cardiovascular risk factors) were associated with higher odds of program completion. Consistently higher odds of completion were observed for individuals with one to five or greater cardiovascular biomarker deficits when compared to those with none, and for individuals with one to five or greater traditional cardiovascular risk factor deficits. This finding is somewhat counter-intuitive as patients with a greater FI value have been shown to be more likely to drop out of CR [25]. When cumulative cardiovascular biomarker deficits were considered, very little difference in the OR estimates for completion existed for individuals with one-five or more deficits when compared to those with no deficits. This is an intriguing finding, as individuals who had accumulated five or more deficits in this category would be presumed to have a greater number of comorbidities and worse health overall, but they had an increased likelihood of program completion. A similar relationship exists in the traditional cardiovascular domain – which is contributed to heavily by the cardiovascular biomarkers. One explanation for this could be that physicians focus on patients with poorer health, as measured by these traditional cardiovascular risk factors. That is, physicians may believe that these patients stand to gain more by participating in CR than their biologically healthier peers; responsible for this could be medicine’s history of treating disease as a biological phenomenon rather than the current – more holistic – biopsychosocial model of disease. On the balance, patients with more health problems may recognize that they can benefit from CR should they complete the program. Findings displayed herein identify many factors play a role in CR adherence, aside from purely biological ones. Physicians may consciously or unconsciously believe that patients with a greater burden of non-traditional CV risk factors will not benefit from the traditional exercise-based CR model, as evidenced by the low rates of completion amongst these patients. However, if these patients were targeted correctly – through both traditional and non-traditional means – they could stand to benefit both mentally and physically from CR.

Lower odds of completion were observed for individuals with a greater number of accumulated body composition deficits, and non-traditional cardiovascular disease deficits. This means that for each additional deficit accumulated in these domains, the likelihood of completion decreases. This finding suggests that these variables, which are not traditionally considered in the context of cardiovascular disease, have an impact on CR completion. Additionally, individuals with eight or more non-traditional cardiovascular risk factors had significantly decreased odds of completion. To our knowledge, this is the first paper to undertake this investigation. These findings highlight the importance of considering non-traditional (eg. Quality of life) variables when prioritizing individuals for interventions that improve retention in CR. These non-traditional variables were mostly related to quality of life as measured by the SF-36, meaning that patients with lower self-reported quality of life are more vulnerable to program dropout. Again, this emphasizes the importance of treating disease holistically and keeping this in mind when delivering CR programs. Increased focus on quality of life could translate into increased rates of CR completion, therefore, decreasing risk of further frailty and/or complications related to CVD. This phenomenon could be investigated in the future by incorporating interventions aimed specifically at improving mental health – such as psychological counselling, or meditation – into CR; therefore increasing the number of health domains targeted by the program. Further, the effectiveness of these interventions could then be examined using a quasi-experimental study design comparing completion before and after the inclusion of these interventions.

When interaction analyses were conducted, we concluded that participant factors had little impact on the associations between FI deficits and CR completion. That is, the relationships between FI contributors and CR completion were not modified by any of the demographic or participant factors that were investigated. This suggests that consideration of frailty deficits is important to maximize CR retention despite participant demographic factors. Additionally, similar strategies for retention can be applied to all participants based on their FI deficits, irrespective of their sex, referring diagnosis, or other demographic variables.

Based on the individual health deficit associations, participants with worse clinical indicators, body habitus, and recent health decline were less likely to complete CR. Many potential factors could contribute to these findings, such as physicians using negative language in consultations with patients, or patients having poorer perception of their health overall based on their physical characteristics. These are examples of factors that could de-motivate patients and alter their likelihood of CR completion. On the other hand, patients with higher quality of life ratings in several categories, and those with higher fat free mass were more likely to complete the program; thus, increased quality of life could positively reinforce and motivate individuals to improve their quality of life.

### Limitations and strengths

There are limitations to be considered with the present study. Regarding selection bias, individuals who did not have sufficient information to calculate an FI could have differed significantly from the remainder of the population, therefore influencing our results. However, these individuals did not differ significantly based on demographic variables, and represented a small minority of the overall sample. A second limitation includes the method by which the accumulation of deficits analysis was completed. If patients had missing values for any of the constituent variables, the maximum number of deficits that could have been identified would be one less than the total number of deficits available. However, all participants considered had enough valid variables to construct a representative FI based on guideline recommendations, which should mean that the risk of bias in this case is low. It is also important to consider that participants in this study reside in a large urban center, which may impact the generalizability of these results. This study also does not capture other reasons why people dropped out of CR; for example, younger people may be less likely to complete CR due to work or family commitments. The current study could also have issues related to multiple comparisons; however, false discovery rate testing did not detect any significant changes in *p*-values for the individual variable analysis (Supplemental Table S4). Additionally, the FI used in the current study did not contain deficits related to other chronic conditions which are known to impact CR completion. Lastly, we could not determine the effect of changes in FI variables with program completion rates as participants did not attend their formal outtake assessment appointment where routine data used in the FI are collected.

Despite these limitations, our investigation advances the knowledge on how frailty contributes to completing CR. This information may be useful for program directors regarding which participants should be targeted with specific interventions for retention. That is, if administrators are aware of the most relevant frailty-related deficits, they can work to retain individuals who would otherwise be likely to drop out of the program. To identify these participants, data would have to be entered at program initiation, with a code written that could identify patients with the deficits discussed in this paper. Program administrators could then target individuals with the highest risk of drop out at program intake, based on their current deficits – both traditional and non-traditional – by offering them additional sessions or learning materials relevant to their current situation. Following this, a matched quasi-experimental study could be undertaken to compare completion rates before and after the addition of this new intervention, with the goal of identifying whether or not it was efficacious.

## Conclusion

This study adds to our knowledge of the importance of understanding frailty deficits with respect to CR completion, demonstrating the association of 16 individual frailty contributors with CR completion. Indeed, we identify specific frailty deficits which should be considered for targeted interventions in the future to retain the maximum number of participants in this CR program.

## Supplementary Information

**Additional file 1: Table S1.** Breakdown of category composition for accumulation of deficits analyses. **Table S2.** Frailty index variables and their respective cut-off values used at cardiac rehabilitation admission and completion. **Table S3.** Investigation of potential interactions between frailty index contributors and age, sex, referring diagnosis, and baseline frailty level. **Table S4.** False discovery rate adjusted p-values for individual frailty deficits.

## Data Availability

The datasets used and/or analysed during the current study are available from the authors upon reasonable request. The data is not publicly available as it is a clinical dataset which contains personal health information, and while patients consented to have their anonymized data used for publication, they did NOT consent to have their data publicly available in a repository for other researchers. Dr. Scott Kehler (scott.kehler@dal.ca) can be contacted for data requests.

## Abbreviations

CR: Cardiac rehabilitation
FI: Frailty index
CVD: Cardiovascular disease
