# Supplementary material for: Contribution of individual and cumulative frailty-related health deficits on cardiac rehabilitation completion
Source: BMC Geriatr. 2023 Jan 20;23:34. doi: 10.1186/s12877-022-03624-0 (PMC9854083; doi:10.1186/s12877-022-03624-0)
Supplement: Supplementary file 1 — Additional file 1: Table S1. Breakdown of category composition for accumulation of deficits analyses. Table S2. Frailty index variables and their respective cut-off values used at cardiac rehabilitation admission and completion. Table S3. Investigation of potential interactions between frailty index contributors and age, sex, referring diagnosis, and baseline frailty level. Table S4. False discovery rate adjusted p-values for individual frailty deficits. [file 12877_2022_3624_MOESM1_ESM.docx]

**Supplemental material**

**Table S1.** Breakdown of category composition for accumulation of deficits analyses.

| FI Contributor | Cardiovascular | Body composition | Quality of Life | Traditional | Non-Traditional |
| --- | --- | --- | --- | --- | --- |
| Systolic blood pressure | X |  |  | X |  |
| Diastolic blood pressure | X |  |  | X |  |
| Pulse rate | X |  |  | X |  |
| Mean arterial pressure | X |  |  | X |  |
| Pulse pressure | X |  |  | X |  |
| Cholesterol | X |  |  | X |  |
| Low-Density Lipoproteins | X |  |  | X |  |
| High-Density Lipoproteins | X |  |  | X |  |
| Triglycerides | X |  |  | X |  |
| Glucose | X |  |  | X |  |
| NYHA | X |  |  | X |  |
| BMI |  | X |  |  | X |
| Waist circumference |  | X |  |  | X |
| Body fat percentage |  | X |  |  | X |
| Fat Free mass |  | X |  |  | X |
| Food frequency questionnaire |  | X |  |  | X |
| METs |  |  | X | X |  |
| Physical Function SF-36 |  |  | X |  | X |
| Mental Health SF-36 |  |  | X |  | X |
| Role physical SF-36 |  |  | X |  | X |
| General Health SF-36 |  |  | X |  | X |
| Energy SF-36 |  |  | X |  | X |
| Bodily Pain SF-36 |  |  | X |  | X |
| Role Emotional SF-36 |  |  | X |  | X |
| Change in health past year SF-36 |  |  | X |  | X |

**Table S2.** Frailty index variables and their respective cut-off values used at cardiac rehabilitation admission and completion.

| FI Contributor | Cut-off Values |
| --- | --- |
| Systolic blood pressure | 0: 90-140 mmHg  1: <90 or >140 mmHg |
| Diastolic blood pressure | 0: 60-90 mmHg  1: <60 or >90 mmHg |
| Pulse rate | 0: 60-99 bpm  1: <60 or >99 bpm |
| Mean arterial pressure | 0:70-110 mmHg  1: <70 or <110 mmHg |
| Pulse pressure | 0: 30-60 mmHg  1: <30 or >60 mmHg |
| Cholesterol | 0: <=6.2 mmol/L  1: >6.2 mmol/L |
| Low-Density Lipoproteins | 0: 0.98-3.36 mmol/L  1: <0.98 or >3.36 mmol/L |
| High-Density Lipoproteins | 0: >= 1.03 mmol/L  1: <1.03 mmol/L |
| Triglycerides | 0: <1.67 mmol/L  1: >=1.67 mmol/L |
| Glucose | 0: 3.9-6.1 mmol/L  1: <3.9 or >6.1 mmol/L |
| NYHA | 0: No shortness of breath  0.33: Some shortness of breath  0.66: Moderate shortness of breath  1: Major shortness of breath |
| BMI | 0= 18.5-25.0  0.5= 25.1-29.9  1= <18.5 or >30 |
| Waist circumference | Women:  0: <=88 cm  1: >88 cm  Men:  0: <=102 cm  1: >102 cm |
| Body fat percentage | Females:  0: <35.6%  0.33: 35.6-40.9%  0.66: 41.0-45.4%  1: >45.4%  Males:  0: <24.3%  0.33: 24.3-28.6%  0.66: 28.7-33.4%  1: >33.4% |
| Fat Free mass | Females:  0: >17.3%  0.33: 15.9-17.3%%  0.66: 14.6-17.2%  1: >14.6%  Males:  0: >20.4%  0.33: 19.1-20.4%  0.66: 17.9-19.0%  1: <17.9% |
| Food frequency questionnaire | 0: >77  0.33: 73-77  0.66: 68.5-73  1: <68.5 |
| METs | 0: >=5 METs  1: <5 METs |
| Physical Function SF-36 | 0: >80  0.25: 60-80  0.5: 40-59  0.75: 20-39  1: <20 |
| Mental Health SF-36 |  |
| Role physical SF-36 |  |
| General Health SF-36 |  |
| Energy SF-36 |  |
| Bodily Pain SF-36 |  |
| Role Emotional SF-36 |  |
| Change in health past year SF-36 | 0: Much better; somewhat better; same  0.5: Somewhat worse  1: Much worse |

**Table S3.** Investigation of potential interactions between frailty index contributors and age, sex, referring diagnosis, and baseline frailty level.

|  | p interaction | | | |
| --- | --- | --- | --- | --- |
| FI contributor | Age | Sex | Dx | Baseline frailty |
| Systolic Blood Pressure | 0.46 | 0.67 | 0.32 | 0.2333 |
| Diastolic Blood Pressure | 0.71 | 0.61 | 0.11 | 0.52 |
| Pulse rate | 0.22 | 0.68 | 0.93 | 0.66 |
| Mean Arterial Pressure | 0.56 | 0.88 | 0.26 | 0.79 |
| Cholesterol | 0.47 | 0.64 | 0.09 | 0.08 |
| High Density Lipoproteins | 0.19 | 0.14 | 0.28 | 0.31 |
| Low Density Lipoproteins | 0.26 | 0.9 | 0.08 | 0.06 |
| Triglycerides | 0.86 | 0.52 | 0.26 | 0.7 |
| Glucose | 0.58 | 0.96 | 1 | 0.46 |
| METs | 1 | 0.32 | 0.44 | 0.1 |
| NYHA** |  |  |  |  |
| Physical function | 0.16 | 0.4 | 0.43 | 0.67 |
| Role physical | 0.99 | 0.89 | 0.4 | 0.62 |
| Bodily pain | 0.99 | 0.67 | 0.43 | 0.13 |
| General Health | 0.24 | 0.83 | 0.07 | 0.93 |
| Energy | 0.11 | 0.26 | 0.29 | 0.77 |
| Role emotional | 0.38 | 0.1 | 0.06 | **0.007** |
| Mental health | 0.77 | 0.58 | 0.23 | 0.5 |
| Food Frequency Questionnaire | 0.53 | 0.8 | 0.43 | 0.49 |
| Body Mass Index | 0.61 | 0.46 | 0.6 | 0.71 |
| Waist circumference | 0.85 | 0.88 | 0.92 | 0.22 |
| Fat Free Mass | 0.54 | 0.27 | 0.28 | 0.21 |
| Body fat | 0.55 | 0.31 | 0.24 | 0.17 |
| Pulse pressure | 0.78 | 0.45 | 0.15 | **0.02** |
| Change past year** |  |  |  |  |

******Indicates non-valid interaction models.

**Table S4.** False discovery rate adjusted p-values for individual frailty deficits.

| Frailty contributor | Raw p-value | BH adjusted p-value^a^ |
| --- | --- | --- |
| Cholesterol | < 0.001 | 0.0003 |
| Low Density Lipoprotein | < 0.001 | 0.0003 |
| Glucose | < 0.001 | 0.0003 |
| Physical Function | < 0.001 | 0.0003 |
| General Health | < 0.001 | 0.0003 |
| Energy | < 0.001 | 0.0003 |
| Food Frequency Questionnaire | < 0.001 | 0.0003 |
| Waist Circumference | < 0.001 | 0.0003 |
| Mental Health | < 0.001 | 0.002 |
| Triglycerides | 0.002 | 0.005 |
| Role emotional | 0.006 | 0.013 |
| Bodily Pain | 0.008 | 0.017 |
| Change in Health Past Year | 0.009 | 0.017 |
| Body Fat Percentage | 0.016 | 0.028 |
| Fat Free Mass | 0.019 | 0.032 |
| BMI | 0.030 | 0.046 |
| METs | 0.124 | 0.183 |
| Pulse Rate | 0.206 | 0.286 |
| High Density Lipoprotein | 0.377 | 0.496 |
| Pulse Pressure | 0.843 | 0.917 |
| Diastolic Blood Pressure | 0.846 | 0.917 |
| Role Physical | 0.866 | 0.917 |
| Systolic Blood Pressure | 0.906 | 0.917 |
| Mean Arterial Pressure | 0.916 | 0.917 |
| NYHA Score | 0.917 | 0.917 |

^a^BH adjusted p-values are those adjusted according to the methods of Benjamini-Hochberg (1995)^26^
